# Evaluation of the efficacy of topically administered imidacloprid + pyriproxyfen and orally administered spinosad against cat fleas (*Ctenocephalides felis*): Impact of treated dogs on flea life stages in a simulated home environment

**DOI:** 10.1186/1756-3305-5-192

**Published:** 2012-09-07

**Authors:** Douglas H Ross, Robert G Arther, Cristiano von Simson, Veronica Doyle, Michael W Dryden

**Affiliations:** 1 Bayer HealthCare, LLC, Animal Health, P. O. Box 390, Shawnee, KS, 66201, USA; 2 Charles River Laboratories, Preclinical Services Ireland, Ltd., Glenamoy, County Mayo, Ireland; 3 Department of Diagnostic Medicine/Pathobiology, Kansas State University, Manhattan, KS, 66506, USA

**Keywords:** Imidacloprid, Pyriproxyfen, Insect growth regulator, Advantage® II, Spinosad, Comfortis®, Dog, Ctenocephalides felis, Cat flea, Simulated home environment, Flea allergy dermatitis

## Abstract

**Background:**

Cat fleas, *Ctenocephalides felis*, are one of the most common ectoparasites infesting dogs and their environments. This study evaluated the efficacy of imidacloprid + pyriproxyfen (PPF) (Advantage® II for Dogs) and spinosad (Comfortis®) against established *C*. *felis* populations in dogs’ simulated home environments.

**Methods:**

Thirty Beagle dogs were randomly assigned to three groups of 10 dogs each and treated twice (Study Days 0 and 28) with imidacloprid + PPF, spinosad tablets, or a negative control (untreated). Dogs were housed individually in controlled simulated home environments capable of supporting the flea life cycle. Flea infestations were established in these environments by infesting each dog with 100 adult cat fleas on Study Days −21, -16 and 1. The impact of the treatments on fleas in the dogs’ environments were assessed by collecting floor mat samples from each simulated home environment, incubating them for 32 days, and counting the number of emerging adult fleas. On Study Days 7, 14, 21, 28, 35, 42, 49 and 56, after collection of the cocoa matting samples, each dog was infested with an additional 5 ± 1 fleas to maintain the environmental infestations. Flea comb counts on dogs were conducted on Study Days 0 (pretreatment) and 63.

**Results:**

From Study Days 7–28, flea infestations in the imidacloprid + PPF environments were significantly lower (p < 0.03) than those in the spinosad environments. Following the second treatment, flea infestations in all the imidacloprid + PPF environments fell to zero for the remainder of the study. In contrast, flea infestations persisted in some of the spinosad environments through the study’s end.

On Study Day 63 all 10 dogs treated with imidacloprid + PPF were flea free, while only one of the 10 spinosad treated dogs was flea free. Flea counts on the other 9 spinosad treated dogs ranged from 3 to 46 fleas/dog (geometric mean = 8.6). A mean of 405 adult fleas/animal were recovered from the control dogs on Study Day 63.

**Conclusion:**

Flea infestations in environments of dogs treated with imidacloprid + PPF declined more rapidly than in those containing dogs treated with spinosad. Flea infestations were completely eliminated by Study Day 56 in environments of dogs treated with imidacloprid + PPF, but persisted through the study’s end in some of environments of dogs treated with spinosad.

## Background

Cat fleas, *Ctenocephalides felis*, are one of the most common ectoparasites infesting dogs and their environments. Flea infestation is the most frequently diagnosed cause of dermatological conditions in dogs, including flea allergy dermatitis (FAD) [1]. The availability of safe and effective flea control products has dramatically improved flea control on pets. However, despite the wide availability of these products, veterinarians and pet owners continue to struggle with FAD, flea infested pets and environments.

Adult cat fleas spend the majority of their life on their hosts. Within 24–36 hours after a blood meal, female fleas begin laying 40–50 eggs per day within the pet’s hair coat. As the pet moves around, eggs roll off with hundreds to potentially thousands of eggs being deposited into the pet’s environment [2]. In optimal conditions eggs will hatch into larvae within 2–5 days. Within 1–2 weeks larvae develop into pupae, and 2–6 weeks later pupae mature into adult fleas capable of infesting a pet and repeating the entire cycle. To effectively eradicate a flea infestation, both adult fleas and the immature flea stages in the environment must be eliminated.

Most pet owners don’t use insecticides year-round and fail to see the first few fleas infesting their animals. They only realize that there is an infestation when their animals are heavily infested, by which time the household is already infested with immature stages. Therefore, the challenge of flea control includes protecting the pet from adult fleas and preventing reinfestations by eliminating immature stages in the environment.

Once a *C. felis* infestation has been established it can take up to 8 or more weeks to eradicate [3-5]. The use of a product or combination of products with activity against both adult and immature fleas can improve control and decrease the time to eliminate the infestation. The insect growth regulator (IGR) pyriproxyfen (PPF), a juvenile hormone mimic, has ovicidal effects in fleas [6]. Pyriproxyfen was recently introduced in combination with imidacloprid (a nicotinoid insecticide that binds to the postsynaptic nicotinergic receptors of insects) for the prevention and treatment of all flea life stages in dogs and cats.

Here we present the results of a controlled efficacy study designed to evaluate the impact of dogs treated with topical imidacloprid + PPF (Advantage® II for Dogs) and oral spinosad (Comfortis®) on cat flea populations in simulated home environments, compared to untreated dogs.

## Methods

### Standards

This study was conducted in accordance with standards of Good Clinical Practice (VICH Guideline 9) and EMEA/CVMP/EWP/005/2000-FINAL0-REV. 2 Guideline for the testing and evaluation of the efficacy of antiparasitic substances for the treatment and prevention of tick and flea infestations in dogs and cats. The study protocol was reviewed and approved by Charles River Laboratories Preclinical Services Ireland Ltd.’s Ethics Committee prior to the start of the study.

### Investigational veterinary products (IVP’s)

Advantage II for Dogs (9.1% imidacloprid + 0.46% PPF; US EPA Reg. Nos. 11556–152, -127, -218 and −130; Bayer HealthCare, Animal Health) is a topical solution administered at a single or multiple spots (depending on dog size and dose volume) to the skin along the dog’s dorsal midline. Advantage II for Dogs is labeled for the treatment of fleas and lice on dogs.

Comfortis (spinosad; NADA 141–277; ELANCO Animal Health) is a chewable tablet, available in five sizes. Tablets are administered orally to dogs based on their weight (minimum dosage 30 mg/kg). Comfortis is indicated for the prevention and treatment of cat flea infestations on dogs.

The IVP’s were administered twice, on Study Days 0 and 28. Dog body weights taken on Study Days −3 and 7 were used to determine the IVP doses administered on these two days, respectively. Administration followed the manufacturers label directions. The untreated controls received no treatment.

### Animals

Forty purpose-bred Beagles (20 males, 20 females; age range, 8 months to 5 years; weight range, 9.4–17.8 kg) were acclimatized and initially evaluated for this study. During acclimatization all dogs were given a physical examination, bathed with a non-insecticidal shampoo and combed to remove any pre-existing flea infestations. No study animals were immunized or dewormed during the acclimatization period. Dogs were observed for general health at least once daily throughout the 34 day acclimatization period.

Animals had not participated in a previous study involving exposure to any ectoparasiticide within 90 days of study onset. During the study no medications were permitted other than the IVP’s, except when prescribed by the attending veterinarians for specific clinical conditions. Animals were fed with an appropriate diet for maintenance, and water was provided *ad libitum*.

### Animal housing/simulated home environments

Dogs were housed in accommodations that complied with accepted guidelines for pen design/floor area, lighting, humidity, temperature, and welfare (including environmental enrichment and social interaction), as required by local and national regulations. During the studies, temperature and ventilation were controlled, and the environment was monitored to maintain an ambient temperature of approximately 20–25°C and a relative humidity of approximately 55–70%.

Dogs were housed in individual pens (1 m X 2 m) containing raised sleeping areas (0.6 m X 0.75 m) with solid wood barriers between the pens. The floor of each raised area was covered with cocoa matting which had been prescreened to ensure it was not treated with any compound that could have interfered with the flea viability.

### Experimental design

This study utilized a randomized block design, with animal gender and pretreatment viable flea egg (larval hatch) counts as the blocking factors. Between Study Days −31 and −24, the 40 dogs were evaluated for their ability to produce viable flea eggs. On Study Day −31, each dog was infested with 100 (± 5) unfed adult *C*. *felis*. On Study Day −27, a maximum of 100 flea eggs were collected from each dog’s pen, placed in petri dishes, and incubated (26°C - 29°C; 62% - 82% relative humidity) for 72 h (± 2 h). Petri dishes were then examined for larval hatch.

On Study Day −21, the 30 dogs producing the highest numbers of viable flea eggs (hatching larvae) were allocated to one of three treatment groups (5 females and 5 males/group): Group A (Advantage II for Dogs); Group B (Comfortis); Group C (untreated control). Within each gender, dogs were ranked in descending order (highest to lowest) based on Study Day −24 egg hatch counts. Animal identification number was used to break ties (highest to lowest ID). The first 3 dogs (highest counts) within each gender, were assigned to Block 1, the next 3 dogs were assigned to Block 2, and so forth, until the final 3 dogs (lowest counts) were assigned to the final Block. The pre-determined randomization schedule reflected the treatment assignments. This randomization procedure was repeated for each gender. The three groups were balanced by gender and ability to support production of viable flea eggs.

### Environmental flea infestations

Cat fleas (*Ctenocephalides felis*) used in this study originated from Charles River Laboratories Preclinical Services Ireland Ltd.’s flea colony. On Study Days −21, -16 and 1, each dog was infested with approximately 100 (100 ± 5; 35-65% female: 35-65% male) recently-emerged, unfed, adult *C. felis* to establish a continuous flea infestation within each simulated home environment. Fleas were applied along the dog’s dorsal midline in the lumbosacral region. On Study Days 7, 14, 21, 28, 35, 42, 49 and 56, after collection of the cocoa matting samples (see below), each dog was infested with 5 ± 1 fleas (male and/or female) as described above.

None of the procedures in this study were expected to result in undue pain, distress, or discomfort to the study animals. However, in the event that the flea infestation on any dog reached a level that resulted in clinical manifestations, e.g., severe FAD, provisions were in place for flea reduction measures on the animal and in the simulated home environment, as well as administration of appropriate medications to provide relief to the dog.

### Assessment of flea infestations

On Study Day 0 (prior to IVP administration), and Study Days 7, 14, 21, 28, 35, 42, 49, 56 and 63, two circular cocoa mat samples (approximately 5.5 cm diameter) were removed from each simulated home environment. These samples were taken from the same locations in each pen on each day. The locations for all cocoa mat samples were randomly selected prior to study initiation. Each sample removed was replaced with a new mat sample of the same size which was fastened in place with screws. Those locations were not sampled again for the remainder of the study.

For all sample collection times, each cocoa mat sample was placed in a labeled, ventilated 250 mL container, to which flea rearing media and sand (1 part flea media: 2 parts sand) were added. Containers were placed in an incubation room (23°C - 31°C; 55% - 90% relative humidity).

Adult fleas emerging from the incubated cocoa mat samples were counted 32 days after sample collection, e.g., adult fleas emerging from Study Day 0 samples were counted on Study Day 32. The remaining counts were performed on Study Days 39, 46, 53, 60, 67, 74, 81, 88 and 95. These emerging adult flea counts were used as the measurement of infestations in the simulated home environments.

On Study Day 0 (prior to IVP administration) and Study Day 63, adult flea comb counts were performed for all dogs. Following Study Day 0 counts, the fleas were returned to the dogs.

### Assessment of IVP efficacy

The geometric means of weekly post-treatment emerging adult flea counts and Study Day 63 adult flea comb counts were used for efficacy calculations. Geometric means provide a measure of the central tendency of the data that minimizes the effects of extreme values. Geometric means were calculated following transformation using a logarithmic method (averaging the transformed values, and converting the average using antilog to represent a geometric mean). Because some counts were zero (0), all counts were modified by adding one (1) to each count prior to logarithmic transformation. Likewise, one (1) was subtracted from the antilog value to meaningfully represent the geometric mean for each group.

Efficacy was calculated for the weekly post treatment emerging adult flea counts and Study Day 63 adult flea comb counts using Abbott’s Formula:

(1) $$\text{\%Efficacy}\;\left(\text{\%Control}\right)=\frac{\text{Geo}.\text{mean flea}\;{\text{count}}_{\;\left(\text{Control}\right)}-\text{Geo}.\text{mean flea}\;{\text{count}}_{\left(\text{Treatment}\right)}}{\text{Geo}.\text{mean flea}\;{\text{count}}_{\left(\text{Control}\right)}}\times 100$$

### Data analysis

Although dogs were grouped by gender and blocked on the basis of egg hatch counts, the “blocking” of animals and the randomization of animals to treatment groups were designed primarily to maintain balance of treatments throughout the study. Consequently, “block” was not included in the statistical analysis.

Transformed counts [log (count + 1)] of emerging adult fleas were analyzed with a repeated measures analysis of covariance including terms for treatment, individual dog pen, study day, and the interaction of treatment and study day. Transformed counts of adult flea emerging from the Study Day 0 (pretreatment) cocoa mat samples were used as the covariate.

SAS PROC MIXED (SAS® Institute, Cary, NC) was used for analysis with the covariance structures ‘AR(1)’ and ‘ARH(1)’ for data collected on equal intervals, or ‘CS’ and ‘CSH’ for data collected on unequal intervals. Results from the model with the smallest Akaike’s Information Criterion were used.

Because the interaction of treatment and study day was significant at the 0.05 level, the treatment groups were compared through the simple effect of treatment for each time point. These simple effect pairwise comparisons were obtained from the treatment X day interaction.

Study Day 63 adult flea comb counts (transformed as noted above) were analyzed using analysis of covariance, including a term for treatment. Transformed Study Day 0 adult comb counts were used as the covariate.

## Results and discussion

### Simulated home environments

**Figure 1:**
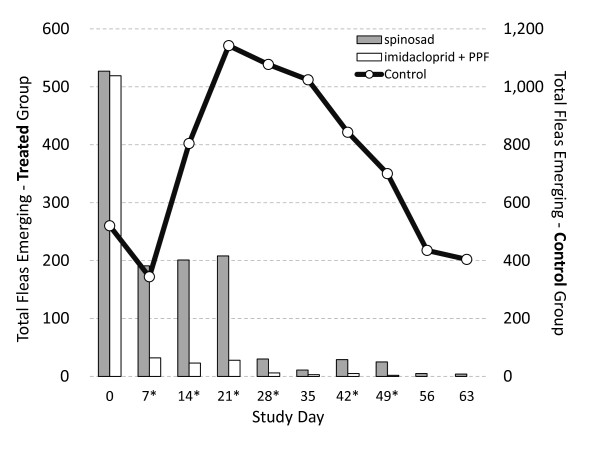
**Total numbers of adult fleas emerging from cocoa mat samples collected from simulated home environments.** The black line shows total numbers of emerging adult cat fleas for untreated controls (right axis). The bars show total numbers of emerging adult cat fleas for spinosad and imidacloprid + PPF treated groups (left axis). On study days marked with an asterisk (*), the differences between emerging flea counts in the imidacloprid + PPF and spinosad groups were statistically significant (p < 0.03).

This simulated home environment model was successful in establishing and maintaining high numbers of fleas throughout the study. Flea infestations (measured by adult flea emergence from cocoa mat samples) were similar for all groups on Study Day 0 (Table 1). Infestations in the untreated controls increased through Study Day 21, and then gradually declined thereafter (Figure 1). This decline was probably due to several factors, including continued removal of (infested) cocoa mat samples, and deterioration of the cocoa mat environment due to contamination with animal waste. In fact, the number of wet and/or soiled cocoa mat samples collected from all environments increased over time. In spite of this decline, infestation levels in the controls on Study Day 63 were still very similar to those on Study Day 0 (Table 1). Furthermore, the high numbers of adult fleas combed from the untreated control animals on Study Day 63 (Table 2) provided additional evidence of the model’s effectiveness.

**Table 1:** Adult cat flea emergence from cocoa mat samples collected from simulated home

		**Study day**									
**Treatment group**		**0**	**7**	**14**	**21**	**28**	**35**	**42**	**49**	**56**	**63**
C (control)	Geometric Means	42.2 ***a***	30.6 ***a***	49.0 ***a***	98.6 ***a***	94.7 ***a***	86.1 ***a***	74.7 ***a***	53.0 ***a***	39.7 ***a***	34.6 ***a***
	Totals	520	344	804	1,142	1,077	1,024	843	700	435	404
B (spinosad)	Geometric Means	47.5 ***a***	14.0 ***a***	15.4 ***b***	17.5 ***b***	2.2 ***b***	0.4 ***b***	1.8 ***b***	1.5 ***b***	0.3 ***b***	0.2 ***b***
	Totals	527	191	201	208	30	11	29	25	5	4
	% Reduction vs. Controls	-	54.1%	68.6%	82.2%	97.6%	99.5%	97.6%	97.1%	99.3%	99.5%
A (imidacloprid + PPF)	Geometric Means	47.7 ***a***	1.4 ***b***	1.3 ***c***	1.7 ***c***	0.3 ***c***	0.2 ***b***	0.4 ***c***	0.1 ***c***	0.0 ***b***	0.0 ***b***
	Totals	519	32	23	28	6	3	5	2	0	0
	% Reduction vs. Controls	-	95.5%	97.4%	98.3%	99.7%	99.7%	99.5%	99.7%	100%	100%

For each study day, means followed by different letters indicate a significant difference between the treatments (p < 0.03).

**Table 2:** Adult cat flea comb counts from control and treated dogs

	**Study day 0**			**Study day 63**		
	**Control**	**Spinosad**	**Imidacloprid + PPF**	**Control**	**Spinosad**	**Imidacloprid + PPF**
Individual Counts From Each Dog	12	7	9	166	0	0
	15	10	17	365	3	0
	21	13	18	389	4	0
	23	26	18	392	5	0
	29	28	21	402	7	0
	31	31	26	422	11	0
	38	38	34	447	18	0
	39	41	52	493	20	0
	49	60	75	514	31	0
	74	71	76	668	46	0
Geometric Means	29.1 ***a***	26.2 ***a***	28.0 ***a***	405.0 ***a***	8.6 ***b***	0.0 ***c***

For each study day, means followed by different letters indicate a statistically significant difference (p < 0.0001) between treatment groups.

### General health and clinical observations

There were no adverse clinical findings immediately (within 24 hours) following administrations of the two IVP’s. Incidences of flea allergy dermatitis (FAD) accounted for most of the abnormal clinical observations recorded during the study. These would be expected in animals that are housed for sustained periods in simulated home environments with active flea infestations. The remainder of the clinical observations could be classified as physical abnormalities that were present in the animals at the start of the study. No animals were removed from the study as a result of any adverse clinical findings.

Seven dogs in the untreated control group were diagnosed with FAD. Three of these cases were first observed between Study Days −6 and −9, while the remaining four were initially diagnosed between Study Days 13 and 57. Four of these seven cases persisted through the end of the study. Three of these persistent cases required treatment with Synulox® Ready-To-Use Suspension (35 mg/mL clavulanic acid as potassium clavulanate and 140 mg/ml amoxicillin as amoxicillin trihydrate) and/or Dexadreson® Injectable solution [2 mg/mL of dexamethasone (as the sodium phosphate)], and one was combed free of fleas on Study Day 48. Three animals in the spinosad group showed signs of FAD. This condition was first observed in two of these animals prior to treatment (Study Days −1 and −3), and on Study Day 27 for the third dog. This latter animal was combed to reduce the number of fleas on Study Day 33. No FAD was observed in any animals in this group after Study Day 33.

One dog in the group treated with imidacloprid + PPF was diagnosed with mild FAD on Study Day −6. This condition persisted throughout the study but required no treatment.

### IVP efficacy in simulated home environments

Imidacloprid + PPF was efficacious (≥ 90% control) in the simulated home environment model from Study Day 7 through Study Day 63, while spinosad was efficacious (≥ 90% control) from Study Day 28 through Study Day 63 (Table 1). Differences in emerging adult flea counts between Group A (imidacloprid + PPF) and Group C (control) environments were statistically significant on all post-treatment collection days (Table 1). Emerging adult flea counts for Group B (spinosad) were significantly different from Group C (control) environments on Study Days 14–63 (Table 1). Emerging adult flea counts for Group A (imidacloprid + PPF) environments were significantly different from those in Group B (spinosad) on Study Days 7, 14, 21, 28, 42 and 49 (Table 1).

After initial administration of the IVP’s (Study Day 0), flea infestations in the imidacloprid + PPF environments declined much more rapidly than those in the spinosad simulated home environments (Figure 1). Flea infestations in the imidacloprid + PPF simulated home environments were > 95% below untreated control levels by Study Day 7 (Table 1). This rapid decline occurred even while flea infestations were still building in the control environments (Figure 1). From Study Day 7 to Study Day 28, flea infestations in the imidacloprid + PPF simulated home environments were significantly lower (p < 0.03) than those in the spinosad simulated home environments (Figure 1).

The insect growth regulator (IGR) PPF probably contributed to this rapid decline. This IGR mimics the action of insect juvenile hormone, preventing both flea egg hatch and development of flea larvae to the adult stage. Flea eggs laid on imidacloprid + PPF treated dogs will not hatch, and the close proximity of the PPF-treated dogs to the cocoa mats may have disrupted development of most flea larvae already present in the mats, preventing their metamorphosis to and emergence as adults.

Following the second IVP administrations on Study Day 28, flea infestations remained relatively low for both treated groups (Figure 1). By Study Day 56, flea infestations in all the imidacloprid + PPF environments were completely eliminated and remained at zero until the study’s end (Figure 1). In contrast, flea infestations were never completely eliminated from all the spinosad environments, persisting in some environments through Study Day 63 (Figure 1).

### IVP efficacy against adult fleas on dogs

On Study Day 0 adult flea counts were very similar for all three treatment groups, with no statistically significant differences (p > 0.30; Table 2). On Study Day 63, infestation of the control dogs was extremely high, with a geometric mean of 405 fleas per animal. As noted above, this demonstrated the effectiveness of the simulated home environment model in supporting the flea life cycle throughout the study.

On Study Day 63, adult flea counts on dogs treated with imidacloprid + PPF and spinosad were markedly lower than and significantly different from the controls (p < 0.0001), and counts on the imidacloprid + PPF treated dogs were significantly lower (p < 0.0001) from counts on those treated with spinosad (Table 2).

All 10 dogs treated with imidacloprid + PPF were flea free on Study Day 63 (Table 2). This IVP effectively eliminated the flea infestations in the simulated home environment and in the dogs. Only one of the 10 spinosad treated dogs was flea free on Study Day 63. Flea counts on the other 9 animals in this group ranged from 3 to 46 fleas/dog with a mean of 8.6. This IVP did not provide complete control of adult fleas, even after two consecutive treatments (Study Days 0 and 28).

While both imidacloprid + PPF and spinosad effectively reduced adult flea infestations in the simulated home environments, imidacloprid + PPF achieved this much more rapidly than spinosad. In addition, by Study Day 56, all simulated home environments of the imidacloprid + PPF treated dogs were completely flea free and remained so through the end of the study. In contrast, all the simulated home environments of the spinosad treated dogs were never completely flea free. A pet owner, when faced with an infested home, wants rapid and complete results in the shortest amount of time. This study indicated the use of topical imidacloprid + PPF could eliminate an infestation faster and more effectively than orally administered spinosad.

Additionally, this study demonstrated the use of either imidacloprid + PPF or spinosad effectively reduced adult flea infestations on dogs compared to untreated control dogs at the end of the study. At Study Day 63, the imidacloprid + PPF treated dogs were flea free, while only one of the spinosad treated dogs was flea free. This is important because a pet owner, using a flea adulticide on their pet, expects 100% efficacy from the product and often has little tolerance for the presence of even a single flea on their pet after several treatments. In this study imidacloprid + PPF was 100% effective in eliminating adult fleas on treated dogs and in their environments.

The IGR activity of PPF likely contributed to the rapid decline of the environmental infestations in the imidacloprid + PPF group after the initial treatment, compared to the spinosad group. While PPF is potently ovicidal [6], the marked decline in emerging fleas within 7 days of initial treatment of the dogs (Figure 1) indicated that the flea larvae in the mats were exposed to PPF and inhibited from molting into pupae [7], which dramatically reduced adult emergence, aiding the rapid elimination of the environmental infestations in the imidacloprid + PPF group.

Previous studies have demonstrated the use of currently licensed veterinary insecticides and insect growth regulators can be effective in reducing or eliminating flea infestations without the use of additional premise treatments [8-10]; however, in cases of severe infestation, additional environmental control measures such as vacuuming, washing pet bedding, application of premise insecticides, etc. may be necessary. It is also prudent for veterinarians to continue to educate clients on the flea life cycle and establish realistic expectations for flea control products when preventing and treating flea infestations.

## Conclusions

Topically administered imidacloprid + PPF effectively reduced adult flea infestations in simulated home environments from Study Day 7 to Study Day 63 inclusive. Orally administered spinosad effectively reduced adult flea emergence from simulated home environments from Study Day 28 to Study Day 63 inclusive

After the initial IVP administrations, flea infestations in the environments of imidacloprid + PPF treated dogs (Group A) declined more rapidly than those in the environments of the Group B dogs treated with spinosad. Following the second IVP administrations, flea infestations in imidacloprid + PPF treated dogs’ environments were eliminated by Study Day 56 and remained at zero through the end of the study. In contrast, flea infestations were never completely eliminated from all the environments of spinosad treated dogs.

Imidacloprid + PPF (100%) and spinosad (98%) IVP’s both effectively controlled adult fleas (*C. felis*) on beagle dogs based on Study Day 63 comb counts. On this day, all 10 dogs treated with imidacloprid + PPF were completely flea free; however, only one of the 10 spinosad treated dogs was flea free. Flea counts on the other 9 spinosad treated dogs ranged from 3 to 46 with a mean of 8.6. The difference in flea counts between imidacloprid + PPF and spinosad treated was significant (p < 0.0001).

The combination of a flea adulticide (imidacloprid) plus an IGR (pyriproxyfen) applied topically to dogs was more effective in reducing and eventually eliminating flea infestations in the dogs’ environments than an adulticide (spinosad) alone. By eliminating the environmental flea infestations, the adulticide + IGR treatment provided superior flea control on the dogs, compared to the flea adulticide treatment alone.

Both imidacloprid + PPF and spinosad were well tolerated.

## Competing interests

DHR, RGA and CvS are employees of Bayer Animal Health, and this work was funded by Bayer Animal Health. VD is an employee of Charles River Laboratories Preclinical Services Ireland, Ltd., where this study was conducted. MWD has served as a consultant and has been sponsored to lecture by Bayer Animal Health and ELANCO Animal Health, manufacturers of Advantage II for Dogs and Comfortis, products that were evaluated in this study.

## Authors’ contributions

DHR was involved in the conception, design and monitoring of the study, interpretation of the data and preparation of the manuscript. RGA and CvS contributed to the study design, interpretation of the data and revision of the manuscript. VD served as the clinical Investigator and participated in the preparation and revision of the manuscript. MWD consulted with the other authors providing expertise on flea efficacy studies, and participated in the preparation and review of the manuscript. All authors read and approved the final version of the manuscript.

## Authors’ information

DHR is a Manager of Clinical Development Projects, RGA is a Senior Research Fellow in Parasitology, and CvS is the Director of Veterinary Technical Services at Bayer HealthCare LLC, Animal Health. VD is a Clinical Investigator/Study Director at Charles River Laboratories, Preclinical Services Ireland, Ltd., Glenamoy, County Mayo, Ireland. MWD is a Professor of Veterinary Parasitology in the College of Veterinary Medicine at Kansas State University.

## References

[B1] Craig M Therapy of flea allergy dermatitis (FAD) in dogs and cats: Part 1 Companion Animal 2012 17 35 39

[B2] Dryden M Host association, on-host longevity and egg production of *Ctenocephalides felis* Vet Parasitol 1989 34 117 122 10.1016/0304-4017(89)90171-4 2588462

[B3] Rust MK Dryden MW The biology, ecology and management of the cat flea Ann Rev Entomol 1997 42 451 473 10.1146/annurev.ento.42.1.451 9017899

[B4] Dryden M Biology of fleas of dogs and cats Comp Cont Ed Prac Vet 1993 15 569 579

[B5] Chin A Lunn P Dryden M Persistent flea infestations in dogs and cats controlled with monthly topical applications of fipronil and methoprene Aust Vet Prac 2005 35 3 89 96

[B6] Palma KG Meola SM Meola RW Mode of action of pyriproxyfen and methoprene on eggs of *Ctenocephalides felis* (Siphonaptera: Pulicidae) J Med Entomol 1993 30 421 426 8459420 10.1093/jmedent/30.2.421

[B7] Stanneck D Larsen K Mencke N An evaluation of the effects of pyriproxyfen on eggs and the cat flea, *Ctenocephalides felis felis* Irish Veterinary Journal 2002 55 8 383 387

[B8] Shanks DJ Rowan TG Jones RL Watson P Murphy MG Smith DG Jernigan AD Efficacy of selamectin in the treatment and prevention of flea (*Ctenocephalides felis*) infestations in dogs and cats in simulated home environments Vet Parasitol 2000 91 213 222 10.1016/S0304-4017(00)00293-4 10940523

[B9] Ritzhaupt LK Rowan TG Jones RL Cracknell VC Murphy MG Shanks DJ Evaluation of the comparative efficacy of selamectin against fleas (*Ctenocephalides felis felis*) infestations on dogs and cats in simulated home environments Vet Parasitol 2002 106 2 165 175 10.1016/S0304-4017(02)00051-1 12031818

[B10] Snyder DE Efficacy of flavored spinosad tablets administered orally to dogs in a simulated home environment (SHE) for the control of existing flea (*Ctenocephalides felis*) infestations [abstract) Proceedings of the 55th Annual Meeting of the AAVP: 31 July – 3 August 2010; Atlanta 2010 51

